# Effects of a tunnel ventilation system within the tie-stall barn environment upon the productivity of dairy cattle during the winter season

**DOI:** 10.5713/ajas.18.0436

**Published:** 2018-09-13

**Authors:** Borjigin Sarentonglaga, Tatsuhiro Sugiyama, Rika Fukumori, Yoshikazu Nagao

**Affiliations:** 1 University Farm, Faculty of Agriculture, Utsunomiya University, Tochigi 321-4415, Japan; 2 Department of Health and Environmental Science, School of Veteruinary Medicine, Rakuno Gakuen University, Ebetsu, Hokkaido 069-8501, Japan

**Keywords:** Winter Season, Tunnel Ventilation, Dairy Cows, Barn Environment

## Abstract

**Objective:**

The objective of this study was to examine the effect of using a tunnel ventilation system within the dairy barn environment upon the productivity of dairy cows during the winter season.

**Methods:**

The study was performed at the University Farm, Faculty of Agriculture, Utsunomiya University. Twenty-one Holstein dairy cows (5 heifers and 16 multiparous) were enclosed in a stall barn. Unventilated (UV) and tunnel-ventilated (TV) was operated by turns every other week, and a number of key parameters were measured in the barn, including tunnel ventilation output, temperature, relative humidity, gas concentrations (oxygen [O_2_], carbon dioxide [CO_2_], and ammonia [NH_3_]). Also, skin and rectal temperature, respiratory rate, blood gas concentrations, and bacterial count were measured from nipple attachments on ten cows. The amount of fodder left uneaten, and general components and somatic cell count of the milk were measured.

**Results:**

As for our dairy barn environment, air temperature dropped significantly with the passage of time with TV. Humidity was significantly higher with TV at 0600 h compared to UV, while CO_2_ and NH_3_ concentrations with UV were significantly higher than with TV at 0000 h and 0600 h. Skin temperature was significantly lower with TV compared to UV at 0000 h and 0600 h. Respiratory rate was also significantly lower at 0600 h with TV than with UV. Bacterial count for the nipple attachments was significantly lower with TV than with UV at 0600 h. The amount of leftover fodder was significantly less with TV in comparison with UV.

**Conclusion:**

Our results suggest that a TV system in the winter barn results in environmental improvements, such as reductions in unfavorable gas concentrations and bacterial growth. Consequently, it is expected that barns utilizing a TV system will be beneficial for both animal health and production.

## INTRODUCTION

Optimization of climatic environmental conditions is important in maintaining health, and in improving the welfare and productivity of livestock. In particular, atmospheric factors such as temperature, relative humidity and air quality (especially the concentrations of NH_3_ and CO_2_) have very important effects upon animal productivity and health [1,2]. During wintertime, it is often the case that all air inlets and outlets of a barn are closed tightly, especially at night, in order to raise ambient temperature. However, without ventilation, barn becomes a closed environment. Consequently, hazardous gases such as CO_2_ and NH_3_ which are derived from feces, urine, eructation, expiration and relative humidity, all inevitably build up considerably. The average of CO_2_ and NH_3_ concentrations ranged 533 to 904 ppm and 7 to 20 ppm, respectively [3,4]. High CO_2_ concentration stimulate the respiratory center and increase respiratory rate, resulted in energy loss. High ammonia concentration in the air causes animal mucosal irritation and tissue inflammations. Prolonged exposure to ammonia can also decrease cow immunity, increase morbidity and reduce milk production [5] and may have a negative effects on the appetite [6]. As a result, this unsanitary and uncomfortable environment may cause detrimental effects upon a cow’s health and productivity.

In recent years, the tunnel ventilation (TV) system has emerged in order to mitigate thermal stress [7] in the summer. TV technology is used extensively for swine and poultry houses, but is relatively new to the dairy industry. A TV system features an air inlet and outlet that are positioned in a straight line. Characterized as having air inlets at one end and exhaust fans at the other [8,9]. In barns, TV systems work to enhance convective heat loss by removing excess heat and humidity from the immediate surroundings of the animals under optimum conditions of the fans, regardless the size of house and the number of cows [10]. In the hot and humid climate of Mississippi, Smith et al [11,12] showed that a free-stall barn equipped with a TV system, and permitting evaporative cooling, reduced the exposure of cows to mid-heat stress, resulting in improved feed intake and milk yield, and reduced milk somatic cell count (SCC), compared to cows exposed to an environment with fans and sprinklers. Nagao et al [13,14] further reported that the TV system prevented increases in both temperature and toxic gas concentrations in the barn environment, and improved milk solids, but not fat content, in dairy cattle during the summertime.

So far, several studies have investigated the effects of TV systems upon the health and productivity of dairy cows in the summer heat, but there are no corresponding reports for winter time. TV systems can be adjusted by altering ventilation fan output, and may therefore be equally effective in maintaining a closed barn environment during the winter. Therefore, the aims of the present study were to investigate the effects of a TV system upon a closed barn environment, and the health and productivity of dairy cattle during the winter period.

## MATERIALS AND METHODS

### Animal care

The procedures used in the present study were performed in accordance with the principles and guidelines for animal use set by Utsunomiya University. All experiments were approved by the Animal Care and Use Committee of Utsunomiya University.

### Housing and animals and management

**Figure 1:**
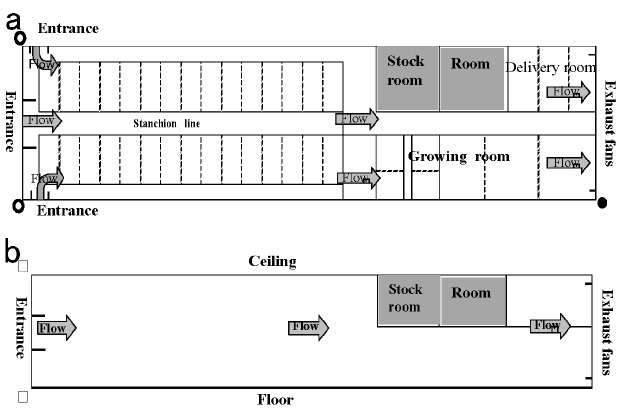
Tunnel ventilation system at the University Farm, Faculty of Agriculture, Utsunomiya University. (a) and (b) represent floor plans of the tunnel ventilation system (width (○—○) : 9 m, length (○—●) : 43 m. The walls of the barn were made of plaster (15 mm thick) or concrete blocks (154 mm thick), while the roof was made form corrugated galvanized irons, asbestos cement plates, and insulating materials (0.4 mm thick) (a). System viewed from above (b) Different sections of the barn (height [□—□]: 5 m). Arrows show images of the direction of air flow from the tunnel ventilation system.

The walls of the barn were made of plaster or concrete blocks, while the roof was made form corrugated galvanized irons, asbestos cement plates, and insulating materials (Figure 1). Holstein dairy cows were managed in individual tie-stalls on a comfortable rubber mat without any bedding within the barn. Water was equipped with water bowls for individual cows. Twenty-one Holstein dairy cows (5 heifers and 16 multiparous) were housed in the experimental barns during the study period. Ten lactating cows (initial day in milk: 73.2±23.1; calving number: 2.0±0.4; initial body weight: 578.6±31.1 kg) in milk were randomly assigned to collect samples. Cows were milked twice daily at 0800 h and 1700 h in tie-stalls using milking unit (Strangko, Denmark). The herd was released to the paddock from 0900 h to 1400 h. At 1400 h, each cow returned to their own sections of the barn every day throughout the experiment. In accordance with the Japanese Feeding Standard (Ministry of Agriculture, Forestry and Fisheries Agriculture, Forestry and Fisheries Research Council Secretariat 1999), cows were fed twice daily at 0600 and 1500 h with 4 kg of corn silage and 1.5 to 6.0 kg of mixed concentrations according to the milk yield and twice daily at 1000 h in a paddock and 1800 at each section of the barn with 4 kg of hay. Fresh water was available at all times from water cup.

### Tunnel ventilation

The TV system used in this experiment was the same as previously described [13]. Briefly, the TV system was closed tightly at night (1800 h to 0600 h), along with the windows and shutters, except the entrance to the ventilation fans consisting of six units (FY-14DGSQR-50; Matsushita Seiko Engineering Co., Ltd., Aichi, Japan, fan capacity: 20,633 m^3^/h) on the north side. Fresh air flowed past the cows before being passing into the exhaust at the far end of the barn. The entrance ratio of the intake openings to the wall surface was set as low as 0.74% (3.48 m^2^) to avoid a reduction in barn air temperature. The rotational speed of the fans was able to set manually or controlled automatically by the temperature sensor [13]. The rotational speed was up to 100% and the amount of airflow was 123,800 m^3^/h when the temperature of the house was 25°C or more.

### Experiment design

Our study was conducted from November to February at the University Farm, part of the Faculty of Agriculture. Firstly, to decide the most suitable output level of the fans in the winter season the outputs level was set to 15%, 20%, and 25%, manually. The amount of airflow was 18,570 m^3^/h at 15%, 24,760 m^3^/h at 20% and 30,950 m^3^/h at 25% of ventilation, respectively. As the volume of the air in cowshed requiring ventilation was 1,624.3 m^3^, the ventilatory frequency was determined to be 11.5, 15.3, and 19.1 per hour. Wind speed, condensation and freezing of water cup were observed at 0600 h in each three days for each output level of TV.

Secondary, in the first one week of the research, barns were unventilated (UV) condition, in the next one week, barns were TV system and turned the condition every week with interval of one week. Each ventilation condition was repeated out three times using same animals.

The UV condition was set to the conventional ventilation pattern of barns in this University farm. All windows and shutters, except the opening of the ventilation fans, were closed at all times in order to close the barn without ventilation.

### Environmental measurement and analyses

**Figure 2:**
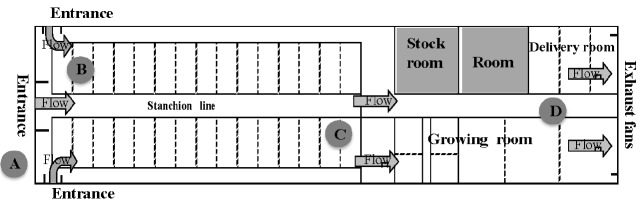
Schematic showing the location of sampling points in the cow shed for wind velocity, temperature and humidity. Sampling point (A) was outdoors, while (B, C, D) were indoors.

Wind speed was measured at a height of 2.1 m using digital wind speed indicator (CW-30; Three Commercial Co., Ltd., Tokyo, Japan) positioned three locations along the barn: near the entrance (Figure 2B), halfway along the barn (Figure 2C), and near the exhaust fans (Figure 2D) at a height of 2.1 m. Using the same locations, we made measurement of temperature and humidity using a dry-bulb thermometer and precision temperature-humidity monitor (Matsuura Seisakusho, Tokyo, Japan) at 1800, 0000 and 0600 h every day. O_2_, CO_2_, and NH_3_ concentrations were measured at a height of 1 m at 1800, 0000 and 0600 h every day (Figure 2C) with a gas harvester (GASTEC GV-100S; Ltd. Gas Tech, Kanagawa, Japan) placed close to the heads of cattle.

### Animal sampling, measurement, and analyses

We measured several parameters from ten lactating cows kept in the same sections of the barn throughout the experiment. Skin, rectal temperatures and respiratory rate were measured at 1800, 0000, and 0600 h at day 2 in each weeks by using surface thermometer (CT-1200; Custom Co., Tokyo, Japan) with a blanking connection, using a clinical mercury thermometer for animals (Matsuda thermometer; Asahi Techno Glass Co., Chiba, Tokyo, Japan), the auscultation of breath sounds in the chest using a stethoscope, respectively. Blood samples were collected from vein at 1800, 0000, and 0600 h at day 4 in each weeks for measurement of blood PH, and gas components (partial pressures of CO_2_ [pCO_2_], O_2_ [pO_2_], and oxygen saturation [sO_2_]) concentrations by using a blood gas analyzer (Stat Profle, NOVA biochemical, Waltham, MA, USA). Nipple attached bacteria were counted the number of colonies after 24 h incubation of agar attached on nipple at 1800 and 0600 h at day 6 in each weeks.

The milk samples obtained from morning milking at 0800 at day 1, 3, 5, and 7 in each week were stored at 4°C until analysis of milk fat, protein, lactose and solids-not-fat (SNF) ratio, SCC using CombiFoss Milkoscan (Foss Electric, Hillerød, Denmark). The milk urea nitrogen (MUN) concentration was calculated using the milk urea value. Milk yield were recorded automatically using milking unit (Strangko, Skjern, Denmark).

Also, we determined the estimated hay intake during night in the barn by feeding a fixed amount of hay the night before the day that measurements were due to be taken (4 kg/cow), and weighing the hay remaining at 0600 h the next morning daily.

### Statistical analysis

Wind speed, temperature, and humidity data within the barn was averaged from data acquired from three locations (Figure 2B, 2C, 2D) for each day. Temporal changes in environmental and animal parameters in the TV systems and UV condition were summarized as means±standard error of the mean, and mean value of the each time and item of the TV systems and UV condition were analyzed using the Student’s t-test. A p-value of <0.05 was considered statically significant.

## RESULTS AND DISCUSSION

### Ventilation conditions

In the TV system, wind speed became progressively greater as output was increased: 0.07±0.02, 0.1±0.04, and 0.4±0.08 m/s respectively with increasing output. Wind speed was still in the UV condition (0.00±00 m/s Table 1). In the UV condition, airflow was minimal with the only access for air being via gaps between the fans (4.45 m^2^); consequently, temperature drops were limited and water troughs remained unfrozen. However, a greater degree of condensation was detected upon the window glass and walls in the UV condition (Table 1) at output levels of 15% and 20% of TV system, this water freezing were prevented. However, a degree of condensation was detected as output was reduced to 15%, indicating insufficient ventilation in these areas (Table 1). Freezing of water troughs was observed at an output of 25%, indicating that these areas were subject to too much ventilation. Given these results, we concluded that an output of 20% was suitable for our tunnel ventilation system in a barn over winter, since condensation and freezing of water bowls was not observed at this ventilation rate.

### Influences upon the dairy barn environment

**Figure 3:**
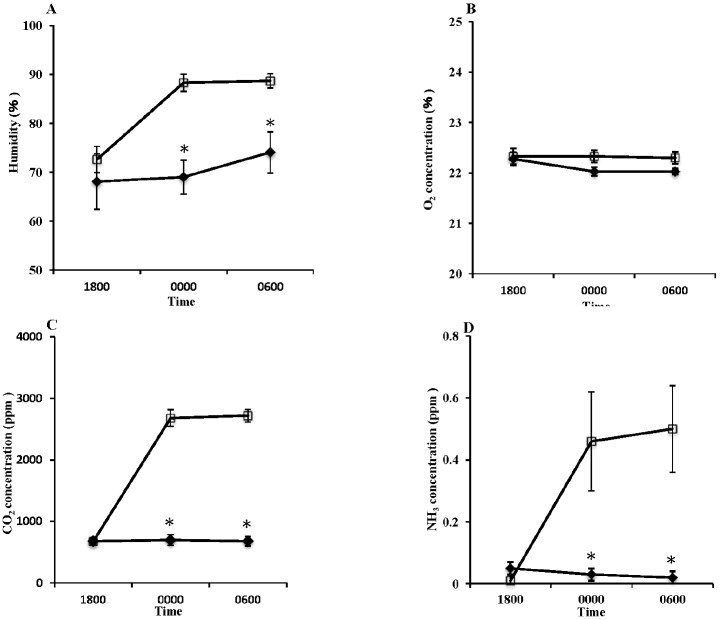
Changes in the dairy barn environment in unventilated (UV □) and tunnel-ventilated (TV ♦) systems. (A) Humidity, (B) O_2_ concentration, (C) CO_2_ concentration, and (D) NH_3_ concentration. Values represent the mean±standard error (n = 21). * Represents statistical difference with respect to TV at p<0.05.

**Figure 4:**
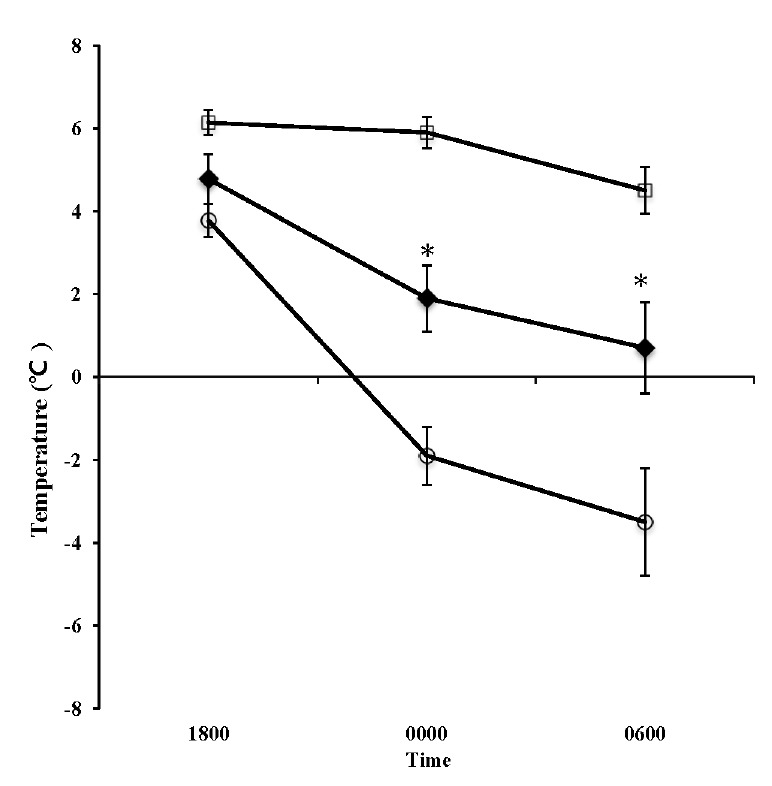
Changes in the dairy barn environment in unventilated (UV □), tunnel-ventilated barn (TV ◆) and the environmental temperature outside of the barn (Outside; ○). Values represent the mean±standard error (n = 21). * Represents statistical difference with respect to TV at p<0.05.

We also investigated changes in the barn environment and the effects of such change upon the cattle. Barn temperature in the TV system and UV condition dropped throughout the night, and the temperature at 0600 h in the TV system was significantly lower than that of UV condition (Figure 3a, p<0.05). Temperature changes were thought to occur in accordance with a drop in external temperatures throughout the night (Figure 4). It has been shown that temperature variations in the range between −0.5°C and +25°C insignificantly affect milk production [15]. The assumed range of neutral temperatures for dairy cattle has been shown −5°C to 25°C [16]. Broucek et al [17] stated that lower critical temperature for cows during peak of milk production was −30°C and cows with average milk yield of 15 kg decrease their milk yield 2 kg in temperatures below −10°C. Since the minimum temperature within the cowshed dropped down to −1°C during TV system operation, the effect of cold stress upon the cattle was thought to be minimal. Barn humidity steadily increased over time in the UV condition (1800: 72.62±2.69, 0000: 88.32±1.73, and 0600: 88.68±1.46) but hardly changed with the TV system (1800: 68.12±3.68, 0000: 69.04±2.47, and 0600: 74.10±2.24) (Figure 3b, p<0.05). We believe that evacuating moist air from within the cowshed and replacing it with an influx of dry air from outside of the shed also prevented a rise in humidity. By preventing such rises in humidity, therefore, the air within the cowshed remained dry and condensation was prevented. As a result, stalls remained dry and it was possible to maintain a sanitary environment.

Changes in the concentrations of CO_2_ and NH_3_ (Figure 2C) close to head of cattle were suppressed during tunnel ventilation with the time lapse (Figure 3d, 3e; p<0.05). Also, there were no differences of the concentration of O_2_, CO_2_, and NH_3_ in any section of the barn (data not shown). CO_2_ is predominantly produced within the barn via cattle exhalation [18], and can be detrimental to cow health. As the concentration of CO_2_ in the air increases, arterial pCO_2_ levels also increase, which upon reaching the medulla oblongata, leads to an increase in respiration, thus increasing CO_2_ emissions [19]. Under normal circumstances, this mechanism maintains normal arterial pCO_2_ levels; however, once CO_2_ levels in the surrounding air reach over 7%, regardless of an increased respiration rate, arterial pCO_2_ suddenly spikes and the body becomes saturated with CO_2_ potentially leading to unconsciousness [20]. On the other hand, NH_3_ forms after microbes break down proteins and amino acids which are present in excreta [18]. Excreta is also the source of malodorous substances such as hydrogen sulfide and methyl mercaptan. These substances quickly become abundant within cowsheds, particularly when ventilation is insufficient. Consequently, they represent a major cause of offensive odors within the shed environment [21]. Furthermore, such cowshed odors can be transferred to the milk via the blood stream after entering the body through respiration, or even directly into bulk milk tanks, potentially leading to a negative effect on flavor [22]. In this study, the number of cows in the barns was small, however TV system can be expected to obtain improvement effects on barn environment regardless of the scale of the barn and the number of cattle [10]. Therefore, restricting the increase of such gases in barns using tunnel ventilation is highly recommended in dairy farms.

### Influences upon the cattle

Skin temperature was significantly lower in the TV system compared to the UV condition at 0000 and 0600 h (p<0.05, Table 2). The decline of skin temperature accompanied a drop in ambient temperature within the cowshed (Figure 3a). The large drop in skin temperatures observed with the TV system was probably caused by a cooling effect that the airflow (0.1 m/s) had upon body surfaces. Rectal temperature showed a tendency to decrease in both the UV condition and TV systems in conjunction with lowering barn temperature. The decrease of rectal temperature was fewer than that of skin temperature. Most homeothermic animals maintain their internal core temperature by constricting their peripheral blood vessels when they were exposure to low temperatures [23]. By doing so, a temperature gradient is created along their four limbs, thus lowering the surface temperature of the body but preventing heat loss from convection. Even in cold environments, an animal’s core temperature can be maintained by an increase in heat production [24]. In particular, the amount of heat generation in milk production by dairy cattle is immense, so lactating cows can still suffering from cold stress. The respiratory rate in the UV condition was significantly higher than that in the TV system (p<0.05, Table 2). At the 1800 h time point, where the first measurements were taken and the cows were still being milked, the cattle remained unsettled from the milking process. Based on the premise that the respiration rate of cattle while resting is 12 to 15 times per minute, we observed that our cattle remained within this range in the TV system at 0600 h, but were above this range during times without ventilation. This can be attributed to air pollutants such as CO_2_ and NH_3_ accumulating within the cowshed during times of non-ventilation, thus reducing the efficiency of respiration, and thus potentially exposing the animals to stress. This could be another influence in the changes of respiration observed here. In terms of blood composition, regardless of large changes to gas concentrations in the air, particularly CO_2_, there were no conspicuous changes observed in either pH, pCO_2_, pO_2_, or SO_2_ in any sections of the barn (Table 2).

**Figure 5:**
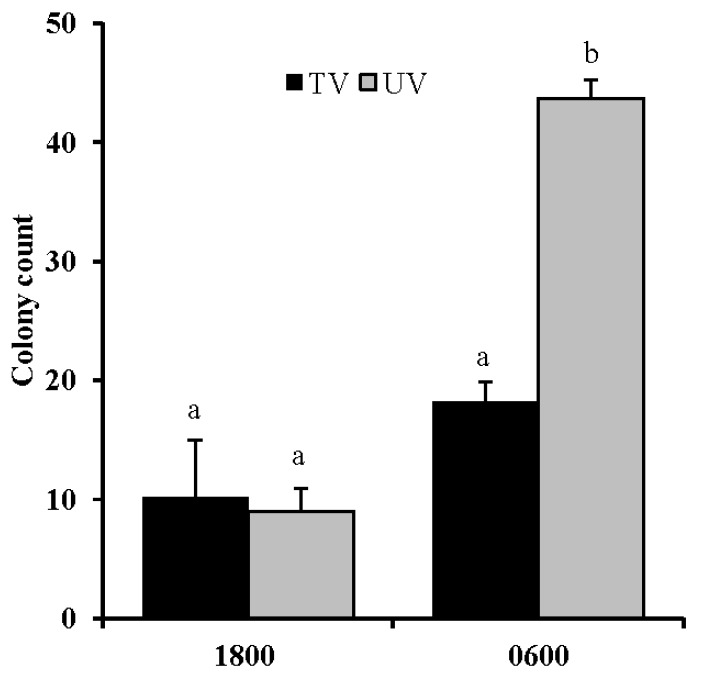
Changes in bacterial count upon nipple attachments in unventilated (UV) and tunnel-ventilated (TV) systems. Values represent the mean±standard error (n = 30). Values with different superscripts (^a,b^) differ significantly (p<0.05).

A large increase in the number of bacteria attached to teats was observed; however, such rises were restricted to when the TV system was being used (p<0.05, Figure 5). This has been attributed to the effect that tunnel ventilation has upon the cowshed environment, where increase of airflow keep the area dry, suppress the replication of bacteria, and assist in maintaining clean teats on the cattle. Such outcomes suggest that a TV system may reduce the risk of environmental mastitis.

### Influences upon the productivity of dairy cows

**Figure 6:**
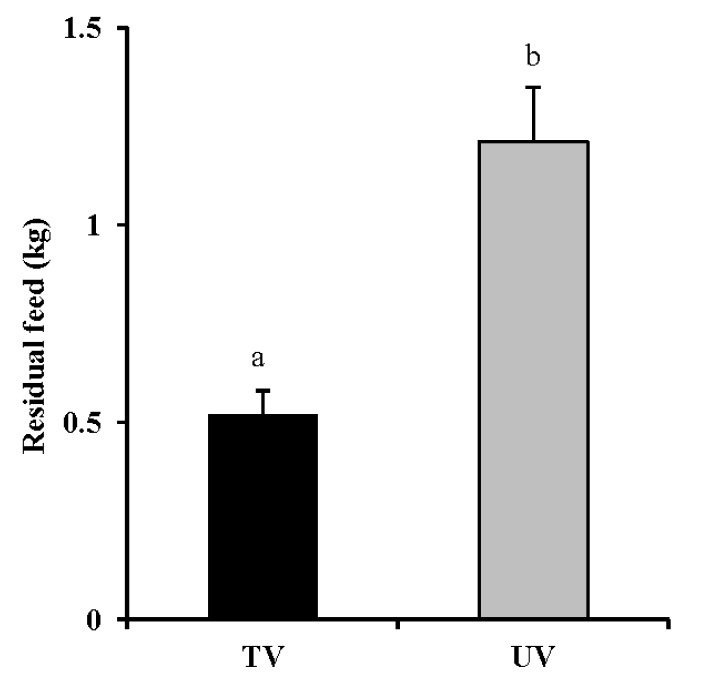
Changes in residual feed in the unventilated (UV) and tunnel-ventilated (TV) systems. Values represent the mean±standard error (n = 21). Values with different superscripts (^a,b^) differ significantly (p<0.05).

So that the volume of leftover feed in the TV system decreased than that in the UV condition the cows in the TV system consumed more feed than that in the UV condition (p<0.05, Figure 6). Dairy cows has been shown comfort zone when environmental temperatures of between 5°C and 25°C [25]. Low relative humidity and harmful gas condition may make promote the appetite of the cows. Or the other hand it was reported that feed intake of the dairy cows increased under the cold condition lower than 5°C [26]. There was no difference in terms of milk fat, protein, SNF ratio, SCC, and MUN, between the TV system and UV condition (Table 3). When changes in milk quality were analyzed throughout each day, however, we observed an increasing proportion of milk fat from cattle in the TV system. Fluctuation of milk fat ratios is predominantly considered to be related to an excess or deficiency in dietary roughage [27]. Mean SCC tended to be lower (p = 0.09) in cattle from the TV system than the UV condition. The high SCC observed during times of no ventilation was thought to be the effect of bacteria present on the teats [11,28,29]. Bacteria attached to the teats has been shown to be able to penetrate into udder from the openings in milk leaking situation before the milking and/or during milking after insufficient washing of the teat [30]. This can lead to bacterial infections in the mammary glands, with the stress of inflammation causing a defense mechanism to activate, which in turn, acts to protect the mammary glands [31,32]. As a result, leukocytes migrate along the capillary walls and the mammary gland cell wall in an amoeba-like fashion, seeping into the milk within mammary gland cells. Once neutrophilic leukocytes infiltrate the milk, they are unable to return to the blood and thus accumulate, increasing SCCs in the milk. At week 3 and 4 of this study period, the onset of mastitis was observed in three cows in the UV condition. As with increased SCC, a bacterial infection of the udder caused by bacteria during leaking and milking present on the teats was thought to be one of the possible causes. In future, in order to further ascertain the effects of TV systems upon productivity, it is necessary to carry out a detailed and extended examination of milk quality, milk yield and pathogenic bacteria of the mastitis preferably over a long time period.

## CONCLUSION

This study suggests that relative humidity and harmful gas in a closed barn during the winter season could be mitigated by using a TV system. This type of ventilation can help keep the barn environment clean and dry, and thus improving the health and productivity of cattle in a practical use, not only in the summer season but also will be applied to the moderate winter climate areas.

## Figures and Tables

**Table 1:** Changes in the dairy barn environment after the overnight staying in the unventilated (UV) and tunnel-ventilated (TV) system

Ventilation system	Output (%)	Wind speed (m/s)1)	Condensation2)	Freezing of water cup2)	External ambient temperature (°C)
UV		0.00±0.00	+++	−	−2.5
TV	15	0.07±0.02	+	−	−3.2
	20	0.1±0.04	−	−	−2.9
	25	0.4±0.08	−	+	−3.3

1) Wind speed were showed mean±standard error (m/s).

2) Condensation and freezing of water cup were showed as below.

−, Represents no condensation; +, Represents minimal condensation; ++, Represents a lot of condensation; +++, Represents significant condensation.

**Table 2:** Physiological parameters of cattle held in the unventilated (UV) condition and tunnel-ventilated (TV) system (n = 30)1)

Items	Ventilation system	Measurement time		

		1800	0000	0600
Cow shed condition				
Skin temperatures (°C)	UV	30.35±0.35	28.26±0.52	26.81±0.46
	TV	30.18±0.4	26.61±0.63*	24.36±0.64*
Rectal temperature (°C)	UV	38.64±0.03	38.44±0.05	38.31±0.14
	TV	38.63±0.04	38.50±0.09	38.23±0.10
Respiratory rate (No./min)	UV	16.67±0.91	16.94±1.15	16.28±0.81
	TV	15.63±0.85	15.21±0.88	13.35±0.74*
Blood component				
PH	UV	7.41±0.01	7.4±0.01	7.43±0.01
	TV	7.41±0.05	7.42±0.01	7.42±0.01
pCO_2_ (mmHg)	UV	34.79±1.38	35.19±1.07	37.48±1.17
	TV	33.78±0.73	35.45±1.00	37.61±0.80
pO_2_ (mmHg)	UV	32.56±0.66	33.09±0.49	31.36±0.38
	TV	31.98±0.65	31.92±0.75	32.69±0.47
sO_2_ (%)	UV	61.34±1.21	61.76±0.82	59.69±1.64
	TV	55.81±1.55	61.68±1.37	61.65±0.85

1) Data represent means±standard error of the mean of ten cows in three experimental periods in each ventilation system (n = 30; 10 cows×1 day×3 terms).

* Represents a significant difference between unventilated (UV) condition and tunnel-ventilated (TV) system (p<0.05).

**Table 3:** Lactation perfomance in the unventilated (UV) condition and tunnel-ventilated (TV) system (n = 30)1),2)

Items	Ventilation system	Measurement date			

		Day 1	Day 3	Day 5	Day 7
Milk yield (kg/d)	TV	20.56±0.92	21.85±0.68	21.36±0.88	22.06±0.32
	UV	19.80±0.32	21.16±0.42	20.85±0.52	21.16±0.82.
Milk fat (%)	TV	3.62±0.33	3.68±0.34	3.59±0.42	4.28±0.33
	UV	3.88±0.54	4.10±0.24	3.85±0.35	3.65±0.18
Protein (%)	TV	3.34±0.15	3.36±0.16	3.36±0.15	3.29±0.17
	UV	3.40±0.13	3.33±0.17	3.39±0.12	3.32±0.15
SNF (%)	TV	8.89±0.08	8.93±0.11	8.89±0.11	8.79±0.04
	UV	8.75±0.15	8.96±0.08	8.94±0.04	8.84±0.08
SCC (cell/mL)	TV	3.48±0.58	3.38±0.78	2.95±0.67	3.40±0.92
	UV	4.75±0.59	4.35±1.16	3.95±1.06	3.85±0.84
MUN (mg/dL)	TV	9.35±0.38	9.45±0.63	10.78±1.06	9.23±0.22
	UV	8.00±0.59	9.35±0.29	11.58±0.65	9.95±0.76

SNP, solids-not-fat; SCC, somatic cell count; MUN, milk urea nitrogen.

1) Data represent means±standard error of the mean of ten cows in three experimental periods in each ventilation system (n = 30; 10 cows×1 day×3 terms).

2) There were no significant differences between the unventilated (UV) condition and tunnel-ventilated (TV) system (p>0.05).
